# Effect of low-dose hydrocortisone and inhaled nitric oxide on inflammatory mediators and local pulmonary metalloproteinases activity in LPS-induced sepsis in piglets

**DOI:** 10.1038/s41598-023-38311-6

**Published:** 2023-07-13

**Authors:** Liliana Kiczak, Urszula Pasławska, Waldemar Goździk, Barbara Adamik, Marzena Zielińska, Stanisław Zieliński, Kacper Nowak, Michał Płóciennik, Jacek Bania, Aleksandra Tabiś, Marcin Nowak, Robert Pasławski, Claes Frostell

**Affiliations:** 1 Department of Biochemistry and Molecular Biology, Faculty of Veterinary Medicine, Wrocław University of Environmental and Life Sciences, Norwida 31, 50-375 Wrocław, Poland; 2 Veterinary Center, Nicoalus Copernicus University in Toruń, 87-100 Toruń, Poland; 3 Department of Internal Diseases and Clinic of Diseases of Horses, Dogs and Cats, Faculty of Veterinary Medicine, Wrocław University of Environmental and Life Sciences, 50-375 Wrocław, Poland; 4 Clinical Department of Anesthesiology and Intensive Therapy, Wrocław Medical University, 50-556 Wrocław, Poland; 5 Department of Food Hygiene and Consumer Health Protection, Faculty of Veterinary Medicine, Wrocław University of Environmental and Life Sciences, 50-375 Wrocław, Poland; 6 Department of Pathology, Faculty of Veterinary Medicine, Wrocław University of Environmental and Life Sciences, 50-375 Wrocław, Poland; 7 Department of Anesthesia and Intensive Care, Karolinska Institutet Danderyd Hospital, 182-88 Stockholm, Sweden

**Keywords:** Physiology, Molecular medicine

## Abstract

Hospital mortality in sepsis varies between 30–45%. It has been shown that administration of inhaled nitric oxide (iNO) and intravenous corticosteroid in a porcine endotoxemia model attenuated the systemic inflammatory response. We explored the anti-inflammatory effect of a double-treatment strategy (iNO + low-dose steroid) on the lungs in a long-term porcine endotoxic shock model. As metalloproteinases (MMPs) are involved in the initiation of multiple organ dysfunction in septic shock, we evaluated the influence of this combination therapy on MMP2 and MMP9 activity and proIL-1β maturation. A shock-like condition was established in 23 animals by continuous infusion of *E. coli* lipopolysaccharide (LPS) for 10 h. Then the animals were observed for 10 h. Twelve pigs received iNO and hydrocortisone (iNO treatment started 3 h after the initial LPS infusion and continued until the end of the experiment). Eleven pigs were controls. Pigs treated with iNO and hydrocortisone displayed less inflammatory infiltrates in the lungs than the controls and a lower level of IL-1β. The proMMP2 was significantly decreased in the iNO and hydrocortisone group. The amount of an active MMP9 (~ 60 kDa) was decreased in the iNO and hydrocortisone group. Total gelatinolytic activity was lower in the iNO and hydrocortisone group. Reduced MMP activity was accompanied by a 2.5-fold decrease of the active IL-1β form (17 kDa) in the pulmonary tissue of iNO combined with hydrocortisone exposed pigs. We demonstrated that in a porcine endotoxemia model the NO inhalation combined with intravenous hydrocortisone led to the attenuation of the inflammatory cascade induced by bacterial LPS. The decrease in pulmonary MMPs activities was accompanied by reduced proIL-1β processing.

## Introduction

This year marks 35 years since the discovery of a role of endothelium-derived nitric oxide (NO) in vascular relaxation^1,2^. It is believed to be one of the major advances in biomedical research in the twentieth century. A number of papers have been published on the role of NO in physiological and pathophysiological processes, also underlying its therapeutic potential^3^. NO is produced by one of three NO synthases (NOS) during conversion of l-arginine to l-citrulline^4^. The NO synthases were named after the tissues in which they were first identified. The neuronal NOS and endothelial NOS are constitutive calcium/calmodulin-dependent isoforms, and inducible NOS (iNOS) is a calcium-independent NOS isoform. iNOS is expressed in macrophages and other tissues following immunological stimulation^5^. NO has been shown to play a role of a mediator/protector of ischemia and reperfusion (I/R) of tissue-mediated injury. NO has also a tissue-protective role, which is accomplished through the physiological attenuation of leukocyte adherence to the endothelium, inhibition of immune defenses and the stimulation of endothelial cell regeneration^4^. In medical practice, NO is supplemented either as an inhaled gas or as an infusion of nitrovasodilators, acting as NO donors. Administration of NO during an ischemic insult has been shown to reduce the reperfusion myocardial^3^ and pulmonary^6^ damage. Frostell et al. demonstrated that inhaled NO (iNO) acts as a selective pulmonary vasodilator^7^. Locally in the lungs, iNO improves oxygenation of the arteries and reduces pulmonary hypertension by selectively relaxing vascular smooth muscle cells in the ventilated areas of the lungs^7,8^. iNO therapy was first registered for the treatment of persistent pulmonary hypertension of newborns following the NINOS trials^9^ in the United States in 1999 and the European Union in 2001^10^.

Inhaled nitric oxide exhibits a systemic effect, such as the attenuation of the inflammatory response in lower extremity ischemia–reperfusion in humans^11^. Moreover, NO inhibits the production of a large number of cytokines in lymphocytes, eosinophils, monocytes and other immune cells, including key inflammatory cytokines^12^. Thus, considering the properties of NO, it seems that iNO could have therapeutic potential in treating medical conditions associated with an exacerbated inflammatory state, such as sepsis. In the most recent ‘Sepsis-3′ consensus definition, sepsis is defined as life-threatening organ dysfunction that is caused by a dysregulated host response to infection, and septic shock as a potential fatal medical condition with persisting hypotension^13^. Despite advances in care, the case fatality rate of sepsis in high-income countries is estimated to be 20%^14^. Early mortality in sepsis is probably predominantly driven by excessive inflammatory reactions^13^. Indeed, iNO was claimed to be effective in lowering mortality in sepsis-related Acute Respiratory Distress Syndrome (ARDS) in an NO responder group^15^. Due to the obvious difficulties in obtaining serial human tissue samples, animal models provide a unique opportunity to study pathophysiological reactions related to sepsis. Bacterial lipopolysaccharide (LPS) (endotoxin) is one of the most effective stimulators of the immune system, and therefore, it has been widely applied in different animal species to study aspects of sepsis^16^. Kang et al.^17^ found using a rabbit model that iNO attenuated LPS-induced acute lung injury and pulmonary inflammation. Interestingly enough, in—porcine endotoxemia models, iNO had a modest effect on the inflammatory response, but iNO combined with steroids attenuated the inflammatory response and almost preserved or restored normal histology of both lung and systemic organs^18,19^. On the other hand, treatment with low-dose (supraphysiological) hydrocortisone revealed some beneficial effects on the pathophysiology of septic shock with small reduction in mortality (APROCCHSS and ADRENAL trial)^20^. Many recent studies indicate that the effect of glucocorticoids in septic patients can be boosted by various forms of combination therapy with other immunomodulators^20^. Thus, the mechanisms by which a combination therapy (iNO + hydrocortisone) exerts an anti-inflammatory effect in the treatment of sepsis needs further investigation.

Based on our earlier experience with a porcine endotoxemia model^19^, we developed a porcine model that enables long-term observation and giving better reflection on the clinical reality of patients with sepsis-induced organ dysfunction^21^. Our goal was to use this animal model to determine how iNO combined with hydrocortisone influences the level of inflammatory mediators, as well as the local pulmonary activity of metalloproteinases (MMPs). We focused on MMPs, as this protein family strongly influences inflammatory processes^22^ and MMP9 is able to process proIL-1β to provide a mature, active form of this main proinflammatory cytokine^23^.

## Methods

### Animals

All animal procedures were in accordance to the *Guide for the Care and Use of Laboratory Animals* as published by the National Institutes of Health^24^, and with ARRIVE guidelines. The study was carried out on 23 domestic piglets (*Sus scrofa*, Polish White breed) with an average body weight of 27 kg. All procedures were performed under anesthesia. The animals were fasted overnight (12-h) with 4-h water restriction prior to the start of the experiment. The study was approved by the Bioethical Committee of the Wroclaw University of Environmental and Life Sciences, Poland. Appropriate sedation and anesthesia were used throughout the experiment. The protocol of the study qualified the animals for a terminal, non-recovery procedure. After surviving the 20-h endpoint, the animals were euthanized by an intravenous (IV) overdose of pentobarbital.

### Anesthesia and instrumentation

The pigs were anesthetized as described previously^25^. In short, a primary anesthesia was induced with an intramuscular injection of zolazepam/tiletamine (4 mg/kg, Virbac) dissolved in medetomidine solution (0.08 mg/kg, Orion). After that, peripheral vein catheterization and an IV bolus of propofol (2–5 mg/kg Fresenius Kabi) was administered, followed by tracheal intubation. The piglets were ventilated in a pressure-controlled mode with self-inspiration initiation (Servo 900C ventilator, Siemens-Elema AB, Solna) at an inspired fraction of oxygen (FiO_2_) of 0.3–0.5 and a PEEP of 5 cm H_2_O. The peak inspiratory pressure was set to keep normoventilation, with pCO_2_ ranging between 5 to 7 kPa and peripheral oxygen saturation above 92%. General anesthesia was maintained by continuous IV infusion of propofol (3–6 mg/kg/h, Fresenius Kabi) and fentanyl (0.8–1.3 μg/kg/h, Polfa) during the entire period of the experiment. Doses of a continuous infusion of anesthetics were titrated to effect, increased during instrumentation and decreased to standard sedative doses for the remainder of the study period.

The instrumentation involved catheterization of an arterial line to the carotid artery (BD Careflow artery catheter^®^, Becton Dickinson) and transabdominal catheterization of the urinary bladder (minimal range laparotomy, Rüsch catheter, Kernen). A central venous catheter (BD Careflow central venous catheter, Becton Dickinson, Singapore) and a balloon-tipped flotation pulmonary artery catheter with a thermistor (PAC) (CritiCath SP5105H TD catheter, Becton Dickinson) were placed via an internal jugular cut-down. After instrumentation, the animals underwent a one-hour recovery period. Baseline data were than recorded (time 0) and initial blood samples were drawn from the arterial catheter.

### Experimental groups

The protocol was designed to compare the effects of iNO in a porcine endotoxemia model. The experiment was run for 20 h. Endotoxemia was induced by continuous IV infusion of LPS from *Escherichia coli* O111:B4 (Sigma-Aldrich) dissolved in sterile water to a final concentration of 2 mg/ml. After baseline measurements, an initial dose of 2.5 µg/kg/h was administered over 1.5 h, then the dose was changed to 0.5 µg/kg/h for the next 8.5 h (a total of 10 h of LPS infusion), followed by a further 10 h of observation. Before instrumentation, the animals were randomized into two groups as follows: 1. control group with standard treatment, 2. iNO + hydrocortisone (iNO + HCT) group with standard treatment, combined with iNO and IV hydrocortisone (25 mg given 3 h after LPS infusion and continued until the end of the experiment—repeated at 8 h and 16 h; the total dose of hydrocortisone was 75 mg).

**Figure 1:**
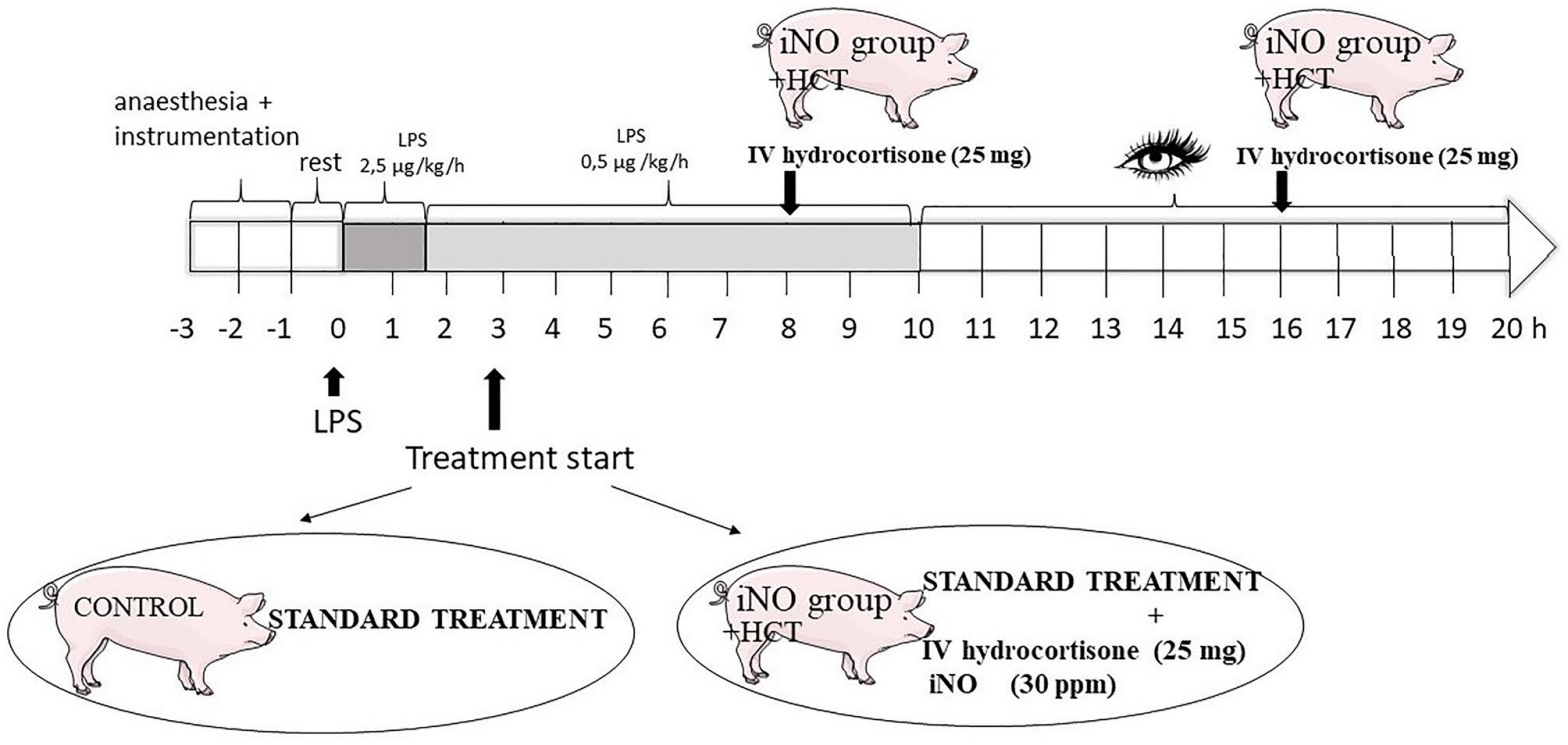
Study protocol. The experiment was run for 20 h. Shock was induced by continuous intravenous (IV) infusion of lipopolysaccharide (LPS). After anesthesia and instrumentation an initial dose of 2.5 µg/kg/h was administered for 1.5 h, then the dose was changed to 0.5 µg/kg/h for the next 8.5 h (a total of 10 h of LPS infusion), followed by a further 10 h of observation. Animals were randomized into two groups: the control group (standard treatment) and the iNO + HCT group (standard treatment + inhaled NO and IV hydrocortisone).

During the experiment, the animals received a 0.9% saline/5% glucose (1:1) at 15 mL/kg/h. In order to reach a PCWP of 8 mmHg the piglets were infused IV boluses of saline if the mean arterial pressure (MAP) was less than 60 mmHg and the pulmonary capillary wedge pressure (PCWP) lower than 6 mmHg. If the PCWP was higher than 8 mmHg and the MAP was lower than 60 mmHg, an IV norepinephrine at 0.025 μg/kg/min was administered and gradually increased targeting a MAP higher than 60 mmHg (up to the maximal norepinephrine dose of 0.6 μg/kg/min). If MAP value exceeded 60 mmHg, norepinephrine was slowly phased out if possible. All piglets received the antibiotic IV cefuroxime (750 mg, GlaxoSmithKline) before instrumentation and the same dose was repeated 8 hourly. A flowchart describing the experimental procedure is shown in Fig. 1. At the end of the experiment the pigs were euthanized using sodium pentobarbital (Morbital, BiowetPulawy) (≥ 100 mg/kg). An autopsy was carried out immediately after euthanasia.

### Administration of iNO

iNO (800 ppm NO in 9000 nitrogen; Pulmonox-Messer Griesheim) was supplied by a Pulmomix Mini (Messer Griesheim) to the inspiratory limb of the ventilator, as reported earlier^26^. iNO (30 ppm) was started 3 h after LPS infusion and continued until the end of the observation period (20 h).

### Monitoring and hemodynamics

The animals were monitored as previously described^19^. Briefly, the following clinical parameters were measured continuously: three-point electrocardiogram (ECG), recordings of heart rate (HR, beats per min), mean systemic arterial pressure (MAP, mmHg), mean pulmonary arterial pressure (MPAP, mmHg), central venous pressure (CVP, mmHg), and the fraction of inspired oxygen (FiO_2_) (General Electric Health Care AS/3 Instrumentarium). Thermodilution cardiac output (CO, L/min) and pulmonary capillary wedge pressure (PCWP, mmHg) were measured every 4 h. Body temperature was monitored with a PAC thermistor, and the piglets were kept normothermic (37–38 °C) using heating blankets or external cooling if needed. The animals posture were changed from side to side every four hours.

### Laboratory specimens

Blood samples for hematological and biochemical measurements and analysis of the arterial blood gases were drawn at baseline (T0, after instrumentation and a 1-h recovery period) and at 4, 8, 12 and 20 h of the experiment (T4, T8, T12, and T20, respectively). On completion of the experiment, tissue sections from the lungs were harvested and frozen in liquid nitrogen. At the same time, separate sections for standard histology were immersed in a 10% buffered formalin solution (pH 7.2) and later embedded in paraffin wax.

### Histology

The 3-μm transverse sections were stained with hematoxylin and eosin (H&E staining) for histopathological evaluation. Slides were evaluated by a veterinary pathologist blinded to treatment groups. Lung specimens were analyzed for the following parameters: passive congestion, red blood cells in the alveoli, inflammatory cell infiltration (lymphocytes, eosinophils, neutrophils, and monocytes), and edema. A scoring system of 0 to 3 was used, with 0 being no change, 1—mild lesion, 2—moderate lesions, and 3—severe lesions, as described in the Supplementary Table S2. To assess the severity of inflammatory cell infiltration in the lungs for each animal, the scores for lymphocytes, eosinophils, neutrophils, and monocytes were pooled and a total score ≥ 2 points was considered heavy pulmonary infiltrates.

### Inflammatory mediators

Lung sections (40–60 mg of each) were homogenized in 300 µl of Tissue Extraction Reagent (Invitrogen) containing the Halt™ Protease Inhibitor Cocktail (Invitrogen). After incubation on ice (15 min) and centrifugation (13,000 rpm, 4 °C), supernatants were collected and stored on ice. Protein quantification was performed using the Bradford reagent (Sigma-Aldrich), according to the manufacturer’s instructions. The amount of inflammatory mediators in the lung homogenates were measured in triplicate with the flow cytometry-based method using magnetic microspheres conjugated with monoclonal antibodies (Luminex xMAP^®^ Technology). Cytokine and Chemokine 9-Plex Porcine ProcartaPlex™ Panel 1 (Invitrogen) allowed for simultaneous detection of IFN-α, IFN-γ, IL-1β, IL-10, IL-12/IL-23p40, IL-4, IL-6, IL-8 (CXCL8), and TNF-α. All measurements were performed on a BioPlex 200 platform with HRF (Bio-Rad), according to the manufacturer's instructions. The data was analyzed using BioPlex Manager 6.0 software (BioRad). The obtained values were normalized using the protein concentrations of each sample. The results are reported as pg/mg protein.

### Gelatin zymography

For the detection of MMP2 and MMP9 proteolytic activity in porcine lung homogenates gelatin zymography was used. In this method, enzyme activity is visible as clear zones in a gelatin-containing gel, where the substrate (gelatin) is digested by enzymes exhibiting gelatinase activity; MMP2 and MMP9 have been shown to specifically digest gelatin^27^.

Lung samples (100 mg) were homogenized in 200 µl of an ice-cold extraction buffer (50 mM Tris–HCl, 200 mM NaCl, 10 mM CaCl_2_, 1% Triton X-100, pH 7.6)^28^. After incubation on ice (30 min) and centrifugation at 9700×*g*, the supernatants were collected and stored on ice. Protein samples (20 μg) were mixed with a non-reducing sample buffer (63 mM Tris, 10% glycerol, 2% SDS, 0.1% bromophenol blue, pH 6.8) and separated at 4 °C on a substrate SDS-PAGE (8% acrylamide, 1 mg/ml gelatin)^28,29^.The gels were washed 3 times in 2.5% Triton X-100 for 30 min and incubated overnight at 37 °C in a collagenase buffer (50 mM Tris–HCl, 200 mM NaCl, 5 mM CaCl_2_, 1 μM ZnCl_2_, 0.2% Brij-35, pH 7.6)^30,31^. Then, the gels were stained using 0.5% Coomassie Blue R-250 (Sigma-Aldrich) in 30% methanol and 10% acetic acid for 60 min and destained in 30% methanol and 10% acetic acid^31^. Gelatinase activity was identified as clear zones against a blue background. Gels were scanned using GelDoc XR (BioRad), and gelatinase band intensity was determined by densitometry, using Quantity One software (BioRad). Individual gelatinase bands were analyzed. The optical density value for the piglet with the lowest activity (from the control group) for a given band was considered as 1; results for all other animals were recalculated accordingly.

### Total gelatinolytic activity

Lung samples (100 mg) were homogenized in 200 µl of an ice-cold extraction buffer (50 mM Tris–HCl, 200 mM NaCl, 10 mM CaCl_2_, 1% Triton X-100, pH 7.6)^28^. After incubation on ice (30 min) and a centrifugation at 9700×*g*, the supernatants were collected and stored on ice. Protein quantification was performed using the Bradford reagent (Sigma-Aldrich), according to the manufacturer’s instructions.

Total gelatinolytic activity was determined using biotinylated gelatin as the substrate. Gelatin (Sigma-Aldrich) was biotinylated using a (+)-Biotin *N*-hydroxysuccinimide ester (Sigma-Aldrich), according to the manufacturer’s instructions. Gelatin-biotin (diluted in 50 mM Tris–HCl, 5 mM CaCl_2_, pH 7.5) was loaded onto a 96 well plate (1 μg/well, Maxisorp, Nunc, Poland) and incubated for 2 h at 37 °C. The plate was washed extensively with PBS containing 0.05% (v/v) Brij-35 (Sigma-Aldrich) and pulmonary homogenates (40 μg) were loaded into the wells and incubated for 24 h at 37 °C in a collagenase buffer (50 mMTris-HCl, 200 mMNaCl, 5 mM CaCl_2_, 1 μM ZnCl_2_, 0.2% Brij-35, pH 7.6)^30,31^. The plate was washed extensively with PBS containing 0.05% (v/v) Brij-35, incubated with streptavidin-HRP (Sigma-Aldrich) for 10 min at room temperature, washed again and developed using the TMB substrate (3,3′,5,5′-tetramethylbenzidine, Sigma-Aldrich). After stopping the reaction, the plate was read at 450 nm. Each sample was measured in triplicate. The mean A_450_ value for the pig with the lowest activity was considered 100%; the results for all other animals were expressed as a percentage of this activity.

### Immunoblotting

Lung samples (30 mg) were homogenized in 200 µl of ice-cold extraction buffer (50 mM Tris–HCl, 200 mM NaCl, 10 mM CaCl_2_, 1% Triton X-100, pH 7.6)^28^ containing a protease inhibitor cocktail at 1:50 (Sigma-Aldrich). The homogenates were incubated on ice (30 min) and centrifugated at 9700×*g*. The supernatants were stored on ice. Protein quantity was determined using the Bradford reagent (Sigma-Aldrich), according to the manufacturer’s instructions. Protein samples (100 μg) were mixed with a reducing sample buffer (Pierce), and incubated at 90 °C for 10 min. The samples were separated using SDS-PAGE in 16% gel and transferred onto a PDVF membrane (Merck Millipore). The membrane was blocked for 1 h with 5% nonfat milk in the PBS containing 0.5% Triton X-100 (Sigma-Aldrich) and incubated overnight with polyclonal goat antibodies against porcine IL-1β (1:1000) (R&D System). Blots were treated with the SuperSignal West Femto ECL (Pierce). The intensities of the bands were determined by Quantity One software (BioRad). The intensity of the target band signal in an individual sample was divided by the intensity of the target band in the internal control (one of the control piglets). The resulting ratios (relative intensities), given as fold change, were used to compare mature IL-1β levels (17 kDa) across the analyzed samples. Each sample was analyzed in triplicate. Glyceraldehyde-3-phosphate dehydrogenase (GAPDH) (1:5000, GeneTex) was used as an internal control for the lung homogenates.

### Statistical analysis

Continuous variables were expressed as the mean and standard error of the mean (SEM); categorical data were expressed as numbers and percentages. All biochemical assessments were performed in triplicate. The Spearman rank test was used for correlations. For the values of inflammatory mediators in lung homogenates and gelatinolytic activity, the Box Cox transformation was used to transform data into normality and the *T*-Student test was used to compare continuous variables between the study groups. For other not normally distributed data, the Mann–Whitney *U* test was used to compare continuous variables between the study groups. The changes in clinical parameters between baseline and the measurement after 4 h of endotoxin infusion were analyzed using a Wilcoxon signed-rank test. Categorical variables were analyzed with the Chi-square test; contingency tables were used to summarize the relationship between several categorical variables. Statistical analysis was performed on the 13.0 version of Statistica (StatSoft, Inc. Tulsa). Values of p ≤ 0.05 were considered to be significant.

### Ethics approval and consent to participate

The animal study was approved by the Bioethical Committee of the Wroclaw University of Environmental and Life Sciences, Poland. All animal procedures and care were conducted in accordance to the *Guide for the Care and Use of Laboratory Animals* as published by the National Institutes of Health^24^. The present study was conducted in accordance with the ARRIVE guidelines.

## Results

### Hemodynamic indices

In the studied model, after 4 h of endotoxin infusion, a marked decrease in cardiac output (from the baseline value of 3.83 ± 0.20 to 2.41 ± 0.17 L/min, p < 0.001), and an increase in heart rate (from the baseline value of 76.95 ± 4.72 to 116.30 ± 8.66 beat/min, p < 0.001) and lactate level (from the baseline value of 2.33 ± 0.25 to 4.10 ± 0.42 mmol/L, p = 0.001) indicated hypodynamic shock and hypoperfusion (Supplementary Table S1). No significant differences were observed between the studied groups throughout the period of the study with the exception of the mean pulmonary arterial pressure (MPAP): MPAP, often observed in shock induced by LPS, was significantly lower in the iNO + HCT group compared to control group after 4 (p = 0.019) and 8 h (p = 0.044) of experiment (Supplementary Table S1).

Animals in both groups were treated with fluids and inotropic support in order to maintain a MAP over 60 mmHg. Additional fluids were administered to obtain filling pressures above 6 mmHg and the procedure was similar in both groups.

### Histopathological findings

**Figure 2:**
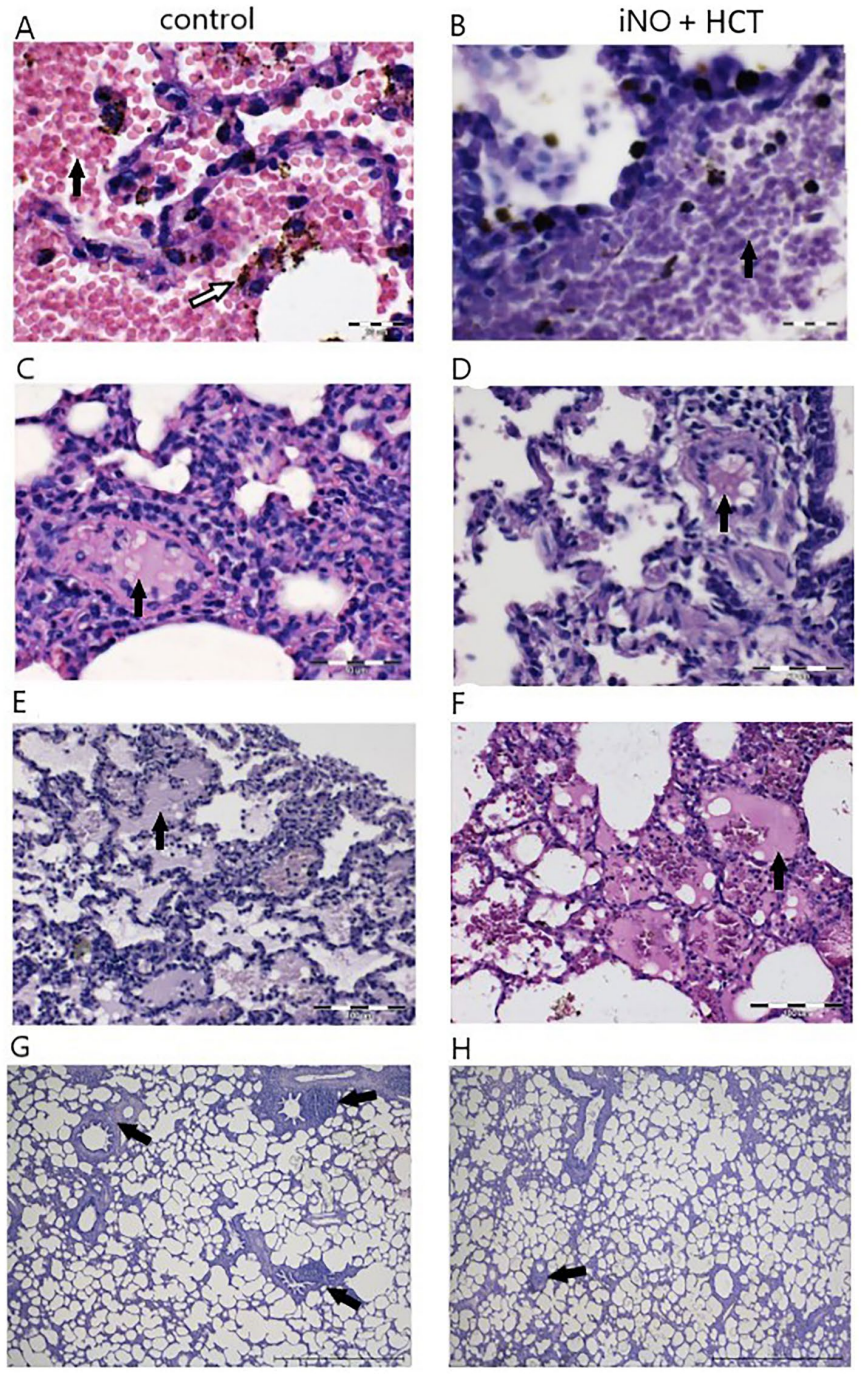
Pigs treated with the combination of iNO and IV hydrocortisone (HCT) displayed significantly less inflammatory infiltrates in lungs than control animals. Representative photomicrograph of lung sections from the control group and iNO + HCT treatment group, stained by the H&E method. Pulmonary congestion (black arrows) in the control group (**A**) and iNO + HCT (**B**) group; note hemosiderin granules (white arrow); globular hyaline microthrombus (black arrows) in the control group (**C**) and iNO + HCT (**D**) group; fluid in the alveolar spaces (black arrows) in the control group (**E**) and iNO + HCT (**F**) group; inflammatory infiltration (black arrows) in the control group (**G**) and iNO + HCT (**H**) group. (**A**,**B**) Original magnification × 1250; (**C**,**D**) original magnification × 800; (**E**,**F**) original magnification × 400; (**G**,**H**) original magnification × 100.

In order to determine the influence of iNO combined with IV hydrocortisone administration on the severity of LPS-induced lung inflammation, lung histology was monitored. In the control group, as well as the iNO + HCT group, light microscopy revealed pulmonary congestion with dilated capillaries and leakage of blood into alveolar spaces resulting in hemosiderin–laden macrophages, indices of immunologically mediated pulmonary hemorrhage (Fig. 2A,B). Some globular hyaline microthrombi in the pulmonary capillaries were found in both analyzed groups (Fig. 2C,D), as well as fluid in the alveolar spaces (Fig. 2E,F). Patchy interstitial inflammatory infiltrates with thickening of the alveolar walls were observed in both animal groups (Fig. 2G,H), as well as formation (activation) of the lymphoid nodules. However, the lungs of pigs treated with iNO + HCT displayed significantly less inflammatory infiltrates than the control animals (p = 0.044). Heavy pulmonary infiltrates including neutrophils, lymphocytes, monocytes, and eosinophils were detected in the majority of the control animals (91%) and only in 50% of the animals in the iNO + HCT group. Mild lesions were found in 86% of the iNO + HCT-treated animals and 14% of the control animals. Semi-quantitative histological data, including inflammatory cell infiltration, red blood cells in the alveoli, and passive congestion are presented in Supplementary Table S2.

### Inflammatory mediators in pulmonary homogenates

**Figure 3:**
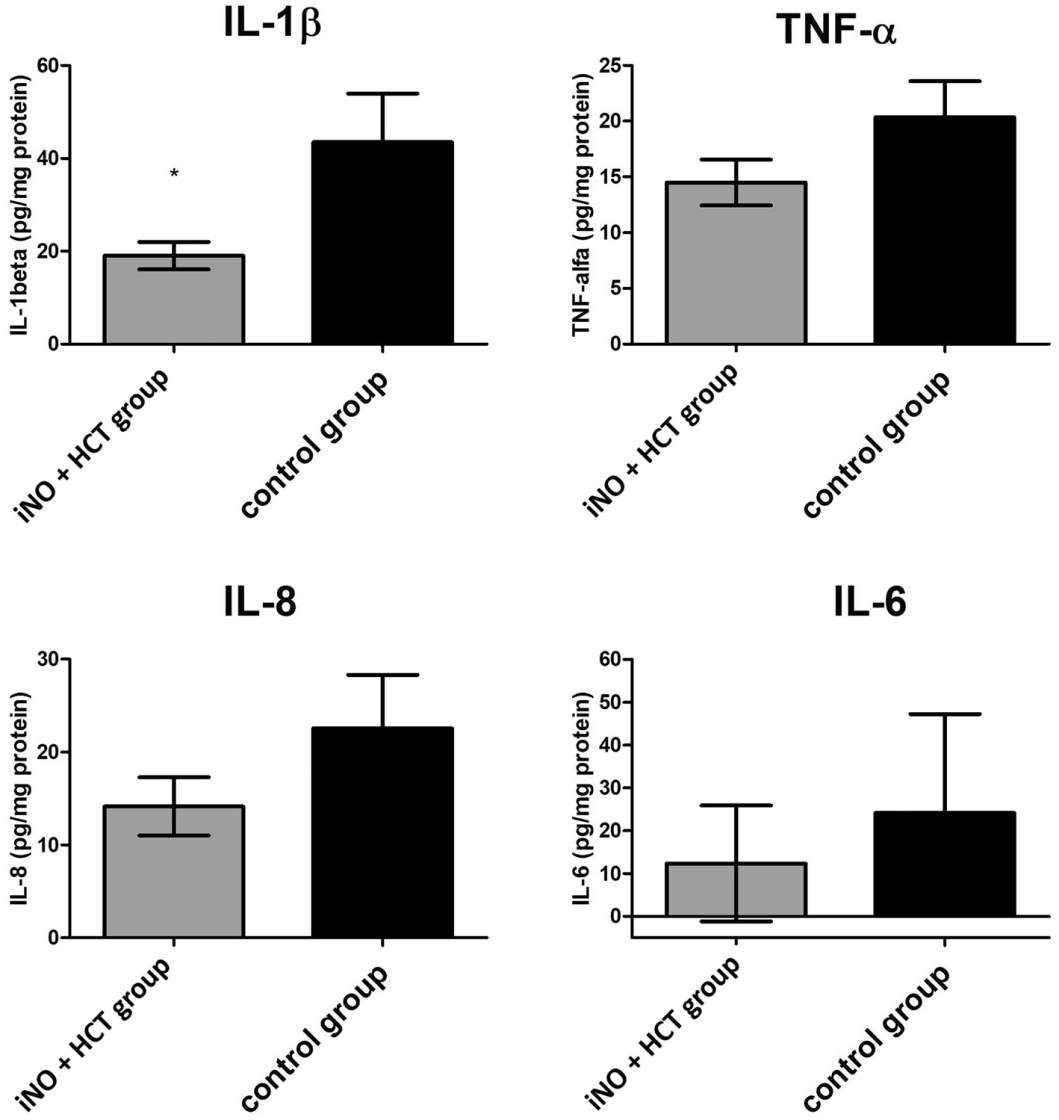
The level of IL-1β is significantly lower in the group treated with iNO and IV hydrocortisone (iNO + HCT) than in the control group. Main proinflammatory mediators in pulmonary homogenates in a porcine endotoxemia model. Values are presented as means ± SEM. Statistical significance was determined by the Mann–Whitney *U* test. *P < 0.05 vs. control group. *HCT* hydrocortisone, *iNO* inhaled nitric oxide, *IL-1β* interleukin-1β, *TNF-α* tumor necrosis factor α, *IL-8* interleukin-8, *IL-6* interleukin-6.

Animals treated with iNO combined with IV hydrocortisone had lower levels of inflammatory mediators in the lung homogenates than the control animals (Fig. 3). However, a statistically significant difference was reached only for IL-1β, with a mean concentration of 19.02 ± 2.93 pg/mg in the iNO + HCT group and 43.45 ± 10.51 pg/mg in the control group (p = 0.029).

### Gelatinases amount and activity in pulmonary homogenates

**Figure 4:**
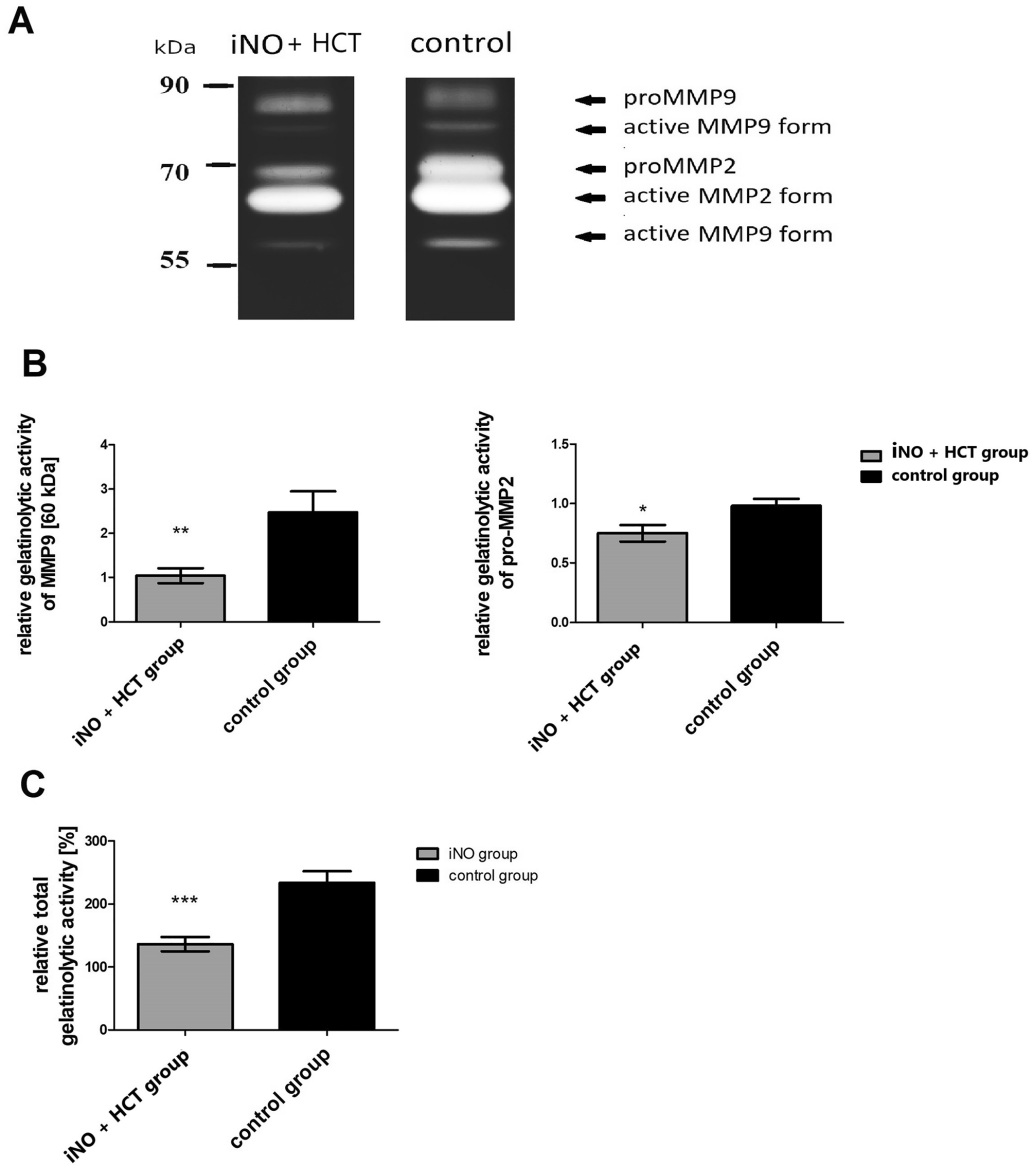
MMP activities are reduced in the pulmonary tissue of iNO and IV hydrocortisone (iNO + HCT) exposed pigs. Gelatinolytic activity in pulmonary homogenates. (**A**) representative gelatin zymograms of pulmonary homogenates from the iNO + HCT group and the control group; densytometric analysis of MMP zymographic activity was performed using pulmonary homogenates from the pigs in the control group and from the iNO + HCT group. The optical density value for the pig with the lowest activity from the control group was set to 1, and then the results for all other animals were calculated accordingly. (**B**) An active form of MMP9 (60 kDa) and proMMP2 (72 kDa); (**C**) The total gelatinolytic activity quantified using a biotin–gelatin assay in pulmonary homogenates from the iNO + HCT and control groups. The measured mean absorbance A_450_ value for a pig with the lowest gelatinolytic activity was considered as 100%; results for all other animals were expressed as a percentage of this activity. Values are presented as means ± SEM. The Mann–Whitney *U* test was used for statistical analysis. *p < 0.05; **p < 0.005; ***p < 0.0005 vs. control group.

Gelatin zymography demonstrated the presence of MMP2 bands (proMMP2, 72 kDa; active MMP2, 68 kDa,) as well as MMP9 bands (proMMP9, 98 kDa; active MMP9 forms, ~ 80 kDa and ~ 60 kDa) (Fig. 4A).The gelatinolytic activities of active and latent forms of MMP2 and MMP9 were quantified. There were significantly lower levels of proMMP2 and the active form of MMP9 (~ 60 kDa)in the iNO + HCT group compared with the control group (Fig. 4B). Other MMP9 bands remained similar in both animal groups (proMMP9, 98 kDa: 1.32 ± 0.18 vs. 1.22 ± 0.22, p = 0.628; active MMP9 form ~ 80 kDa: 2.26 ± 0.48 vs. 2.14 ± 0.63, p = 0.578, in the iNO + HCT group and in the controls, respectively). The total gelatinolytic activity in all the samples was measured using biotinylated gelatin as a substrate. This activity was significantly lower in the iNO + HCT group compared to the control group (136 ± 11 vs. 234 ± 18%, p = 0.0001) (Fig. 4C).

### IL-1β maturation in lung homogenates

**Figure 5:**
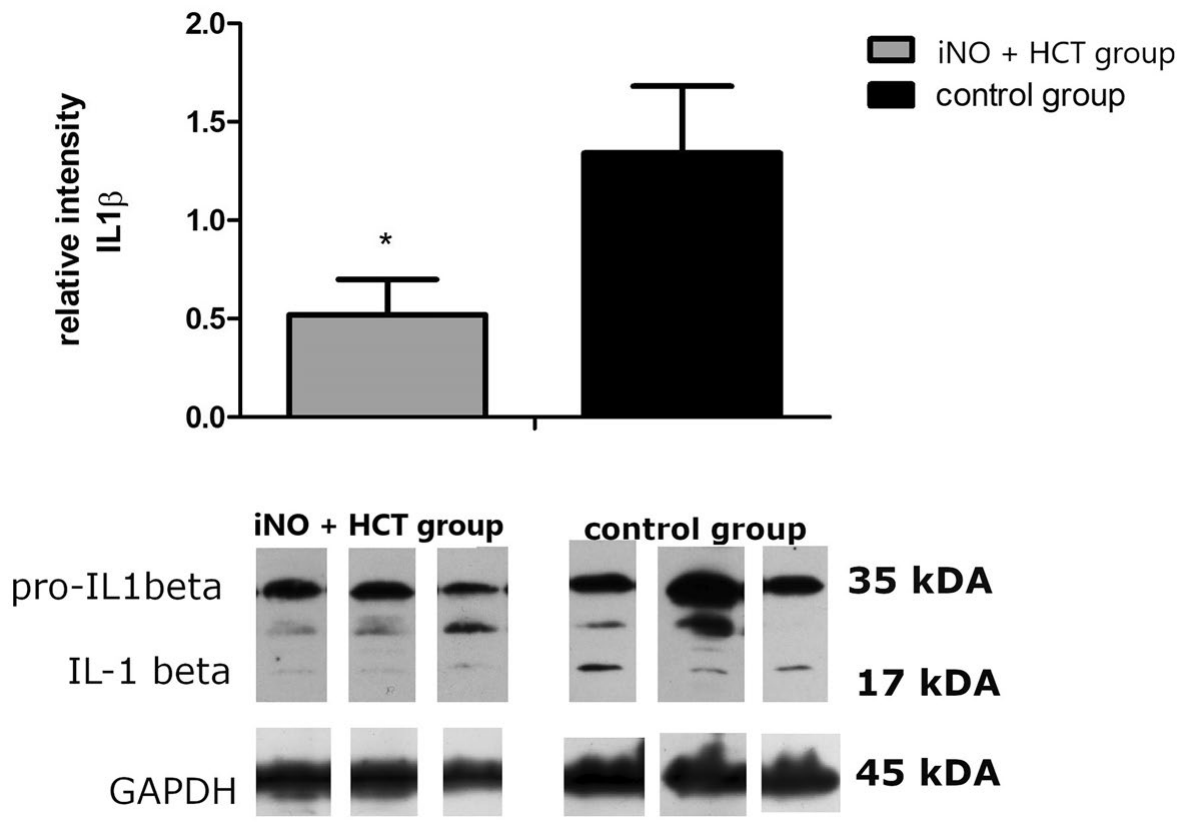
The active IL-1β form (17 kDa) decreases in the pulmonary tissue of iNO and IV hydrocortisone (iNO + HCT) exposed pigs. Densitometric analysis of the mature IL-1β band (17 kDa) in pulmonary homogenates from the control and iNO + HCT groups. The optical density value for the same pig as used in the analysis of gelatinolytic activity (from the control group) was set to 1, and then the results for all other animals were calculated accordingly. Representative Western blots using pulmonary homogenates from a pig from the iNO + HCT group and the control group show 33 kDa proIL-1β band and a mature (active) 17 kDa IL-1β band. GAPDH was used as a loading control for protein normalization. Values are presented as means ± SEM. The Mann–Whitney *U* test was used for statistical analysis. *p < 0.05 vs. control group.

To explore the pro-IL-1β processing (33 kDa proIL-1β is cleaved at Asp116–Ala117, delivering the C-terminal, mature form of IL-1β), lung homogenates were analyzed using Western blot. The mature (active) IL-1β band (17 kDa) was approximately 2.5 times weaker in the iNO + HCT group than in the control group (0.51 ± 0.18 vs. 1.33 ± 0.33, p = 0.038) (Fig. 5), whereas proIL-1β remained at a similar level in both analyzed groups.

### Association between the amount of pulmonary IL-1β and its mature form (17 kDa) with MMPs gelatinolytic activity

On the basis of the obtained data we supposed that by decreasing MMPs gelatinolytic activity, iNO plus low-dose hydrocortisone might influence the amount and processing of the main proinflammatory protein, IL-1β, in the lungs. Thus, correlation analysis was performed to detect possible associations of the concentration of IL-1β in the pulmonary homogenates and a mature IL-1β form (17 kDa) with MMPs gelatinolytic activities. IL-1β was positively correlated to the total gelatinolytic activity (Table 1). The mature form of IL-1β was positively related to the active form of MMP9 (~ 60 kDa) as well as to total gelatinolytic activity.

**Table 1:** Correlation between IL-1β in tissue homogenates and relative intensity of the mature IL-1β and gelatinolytic MMP activities.

	IL-1β		Mature IL-1β	
	r	p	r	p
proMMP2 (72 kDa)	0.194	0.373	**0.414**	**0.049**
Active form of MMP2 (68 kDa)	0.140	0.523	0.336	0.117
proMMP9 (98 kDa)	0.231	0.288	0.212	0.330
Active form of MMP9 (~ 80 kDa)	0.094	0.666	0.219	0.314
Active form of MMP9 (~ 60 kDa)	**0.535**	**0.008**	**0.429**	**0.040**
Total gelatinolytic activity	0.326	0.128	**0.498**	**0.015**

*IL-1β* interleukin 1beta, *MMP2* matrix metalloproteinase 2, *MMP9* matrix metalloproteinase 9. The Spearman rank test was used for correlations. Significant values are in bold.

## Discussion

We have demonstrated that after an endotoxin challenge pigs treated with a combination of iNO and corticosteroid displayed significantly less inflammatory infiltrates and a lower level of a major proinflammatory mediator, IL-1β, in the lungs than in control animals. As lower gelatinolytic activity was accompanied by decreased maturation of proIL-1β in pulmonary tissues in iNO-exposed pigs, we suppose that NO inhalation combined with intravenous hydrocortisone might lower pulmonary MMP9 activity, and as a result, decrease the amount of the mature form of IL-1β, as well as inflammatory infiltrates in the lungs. Our findings suggest that NO inhalation combined with intravenous hydrocortisone might exert an anti-inflammatory effect in sepsis therapy.

Sepsis is an aggressive and multifactorial disease state resulting from the dysregulated host response to infection, leading to multiple organ failure^32^. The lungs are one of the most affected organs^33^. As MMPs have considerable influence on the process of inflammation^22^ and in view of in vitro (steady-state NO exceeding levels of 1 µM leading to the inactivation of MMP9)^34^ and in vivo (NO bioavailability modulating the activity of MMP2 and MMP9 in pregnancy) data^35^, we decided to analyze how iNO would influence the MMP activity in the pulmonary tissue. As air is inhaled directly and quickly in the lungs, this is an ideal place where NO could interact directly with MMPs and reduce their activities. Our results supported this hypothesis. As both MMP2 and MMP9 have been shown to specifically digest gelatin^27^, we used two methods to analyze their activities: zymography (enzyme activity is visible as clear zones in a gelatin-containing gel) and a total gelatinolytic activity assay using biotinylated gelatin as the substrate. The amount of an active form of MMP9 (~ 60 kDa) as well as the total gelatinolytic activity was significantly lower in the animals treated with iNO. Martin et al.^36^ recently outlined the role of MMPs in sepsis pathophysiology: bacterial endotoxin stimulates the release of cytokines that contribute to the expression and release of MMPs; MMPs activate cytokine precursors in a self-perpetuating vicious circle. iNO may attenuate this self-perpetuating vicious circle and the inflammatory cascade induced by bacterial LPS by lowering the activity of MMPs. This assumption is confirmed by histopathological findings: the lungs of pigs treated with iNO displayed less inflammatory infiltrates than the control animals. Moreover, pulmonary homogenates after iNO treatment contained less IL-1β, the main proinflammatory cytokine. We also found that the processing of proIL-1β into the mature and active Il-1β was lower in the lungs in the iNO group, and this phenomenon was related to lower MMP activity. This is in accordance with the finding that proIL-1β maturation can be accomplished by MMP9^23^. MMP9 is believed to be a key molecule in inflammation^37^, i.a. responsible for the proteolytic cleavage of IL-8, IL-1β, and CXCL6, which directly enhances leukocyte recruitment to injured pulmonary tissue^38^. Gerber et al. have recently demonstrated that inhaled broad spectrum MMP inhibitor delivery attenuates pulmonary injury in an endotoxin lung injury model^39^, which is consistent with our results. Thus, it seems that inhibiting MMP activity with iNO might help to attenuate the inflammatory cascade induced in sepsis. Our research focused on iNO influence on the inflammatory process via inhibition of MMP9 activity. However it should be pointed out that in all pigs treated with iNO another anti-inflammatory factor, i.e., hydrocortisone was used. A double-treatment strategy (iNO plus low-dose steroid) was successfully applied in experimental septic shock studies^18,19^. For this, in order to follow the 3R rule—Replacement, Reduction, and Refinement we employed this strategy instead of studying separate effects of iNO and steroid on animals.

To our knowledge, our study is the first insight into the possible mechanism by which iNO combined with intravenous hydrocortisone exerts an anti-inflammatory effect using a porcine endotoxemia model. Our approach ensures accessibility to pulmonary tissue samples and provides a sufficient amount of biological material for biochemical and histological analysis that is obviously not available in the case of humans. As pigs are physiologically and anatomically very similar to humans^16^, and their immune responses resemble humans^40^, research based on our porcine model of endotoxin-induced shock allows for highly probable translation from the laboratory into clinically relevant applications in humans. The intravenous continuous LPS infusion for 10 h used in our model imitates clinical endotoxemia, in which endotoxin remains in circulation for a longer period^16^. We used a relatively low dose of LPS (an initial dose of 2.5 μg /kg/h for 1.5 h, followed by 0.5 μg/kg/h for the remaining 8.5 h) as pigs—similar to humans—are rather sensitive to the administration of LPS (a dose of 25 μg/kg of LPS can be considered high for pigs)^16^. All the pigs were kept continuously sedated throughout the experiment, which counteracted social and handling stress, and activated the hypothalamic-pituitary adrenal axis influencing the immune system^16^. Moreover, the comprehensive care of the pigs (including i.a. mechanical ventilation, vasopressor support, fluid and electrolyte management) was identical to that provided to humans in an intensive care unit. In all important studies where steroids were used in sepsis or septic shock, a total daily dose of 200 mg was given, i.e. approx. 3–3.5 mg/kg, this dose is also suggested in the SSC 2021 Guidelines^20,41^. Taking into account the weight of the experimental animals, a total dose of 75 mg was used, in divided doses of 25 mg in similar time sequences, as is usually used in clinical medicine. The extended time between the end of LPS infusion and euthanasia (10 h) enabled us to evaluate the long-term effect of iNO combined with intravenous hydrocortisone on the pulmonary inflammatory status.

While the number of experimental animals under investigation may seem low, it is nevertheless in good line with studies using similar porcine models^16^. A drawback of experimental pig models (compared to rodents) is cost effectiveness.

## Conclusions

Our study showed that inhaled NO combined with intravenous hydrocortisone attenuated the inflammation process induced by bacterial LPS by lowering pulmonary MMP activity in a porcine endotoxemia model. Recently, Lotz et al.^42^ demonstrated that the utilization of iNO is useful in COVID-19–induced moderate to severe acute respiratory distress syndrome. Our results might suggest that besides providing a positive effect on arterial oxygenation, a therapeutic option of a combination of inhaled NO and IV-applied steroids could inhibit the cytokine storm that develops in patients with severe pulmonary disease, e.g. septic shock and COVID-19^43^.

## Supplementary Information

Supplementary Figure 1.

Supplementary Tables.

## Data Availability

The data that support the findings of this study are available from the corresponding author upon reasonable request.

## Abbreviations

CO: Cardiac output
HR: Heart rate
iNO: Inhaled nitric oxide
IL-1β: Interleukin-1β
IV: Intravenous
LPS: Lipopolysaccharide
MAP: Mean arterial pressure
MMP: Metalloproteinase
MPAP: Mean pulmonary arterial pressure
NO: Nitric oxide
NOS: Nitric oxide synthase
PCWP: Pulmonary capillary wedge pressure
SEM: Standard error of the mean
