# Supplementary material for: Effect of low-dose hydrocortisone and inhaled nitric oxide on inflammatory mediators and local pulmonary metalloproteinases activity in LPS-induced sepsis in piglets
Source: Sci Rep. 2023 Jul 13;13:11369. doi: 10.1038/s41598-023-38311-6 (PMC10344886; doi:10.1038/s41598-023-38311-6)
Supplement: Supplementary file 1 — Supplementary Figure 1. [file 41598_2023_38311_MOESM1_ESM.pdf]

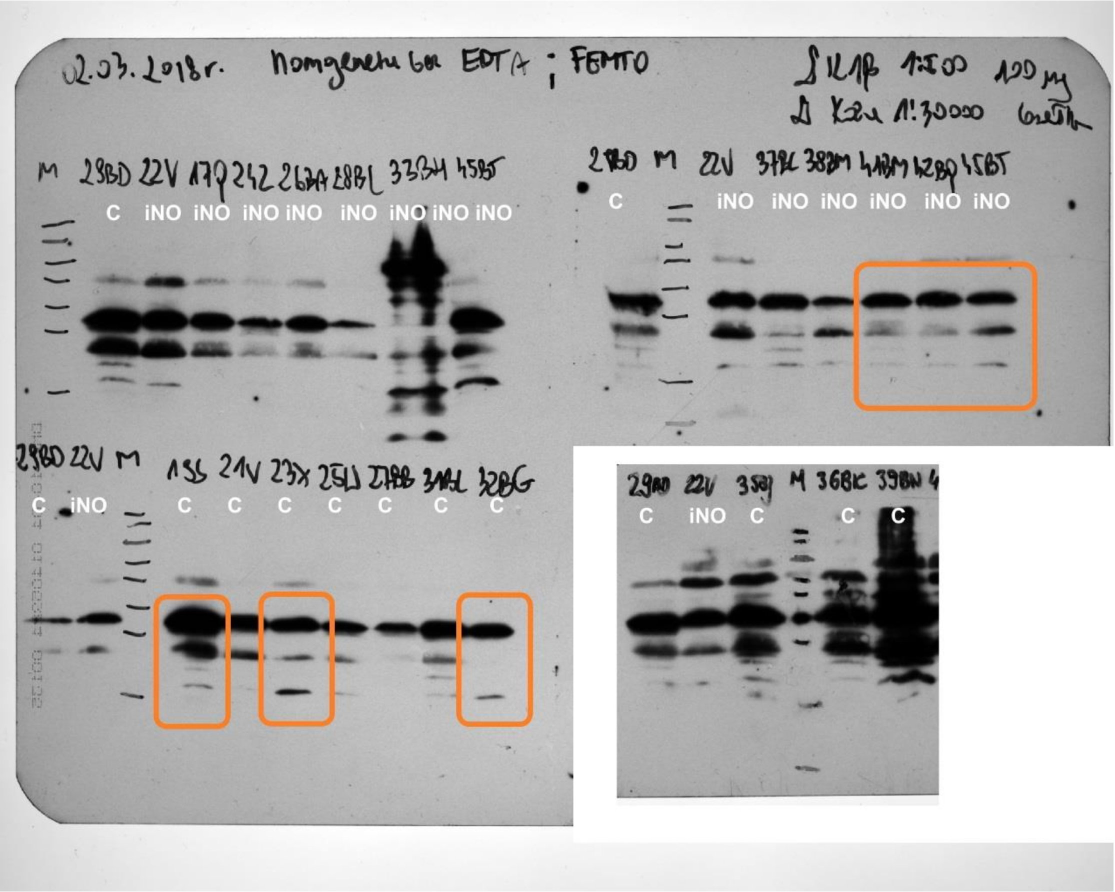

Supplementary Fig. 1. A. Full-length Western blots of pulmonary homogenates from all pigs from the iNO-treated group (iNO) and the control group (C) used in the experiment. The homogenate proteins were resolved in SDS-PAGE, transferred onto PVDF membrane and blotted for IL-1 β. Appropriate lines representing blots of iNO-treated or control animals delineated with frames were used in Fig. 5

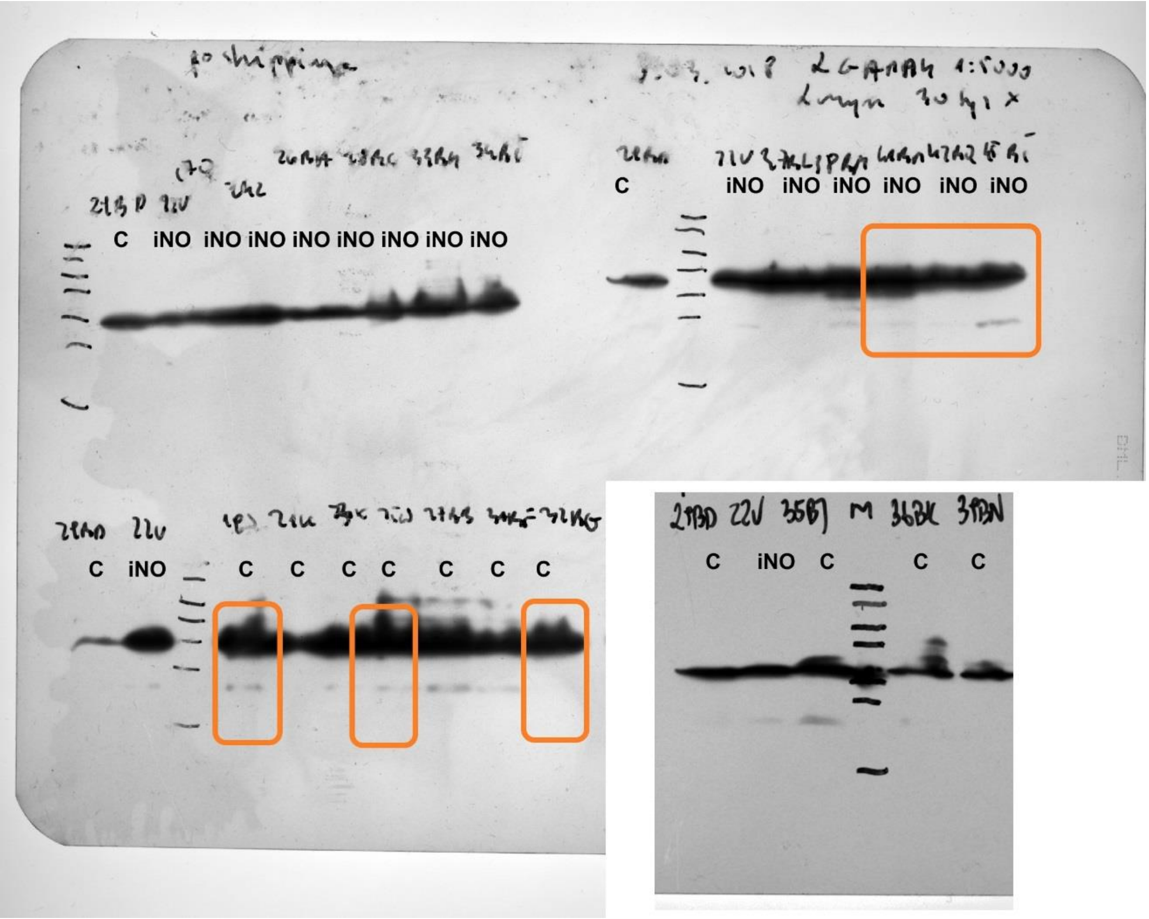

Supplementary Fig. 1. B. Full-length Western blots of pulmonary homogenates from all pigs from the iNO-treated group (iNO) and the control group (C) used in the experiment. The homogenate proteins were resolved in SDS-PAGE, transferred onto PVDF membrane and blotted for GAPDH. Appropriate lines representing blots of iNO-treated or control animals delineated with frames were used in Fig. 5
