# Supplementary material for: Effect of low-dose hydrocortisone and inhaled nitric oxide on inflammatory mediators and local pulmonary metalloproteinases activity in LPS-induced sepsis in piglets
Source: Sci Rep. 2023 Jul 13;13:11369. doi: 10.1038/s41598-023-38311-6 (PMC10344886; doi:10.1038/s41598-023-38311-6)
Supplement: Supplementary file 2 — Supplementary Tables. [file 41598_2023_38311_MOESM2_ESM.docx]

Supplementary Information

Title: Effect of low-dose hydrocortisone and inhaled nitric oxide on inflammatory mediators and local pulmonary metalloproteinases activity in LPS-induced sepsis in piglets.

Liliana Kiczak ^1^*, Urszula Pasławska ^2,3^, Waldemar Gozdzik ^4^, Barbara Adamik ^4^, Marzena Zielinska ^4^, Stanisław Zieliński ^4^, Kacper Nowak ^3^, Michał Płóciennik ^1^, Jacek Bania ^5^, Aleksandra Tabiś ^5^, Marcin Nowak ^6^, Robert Pasławski ^2^, Claes Frostell ^7^

^1^ Department of Biochemistry and Molecular Biology, Faculty of Veterinary Medicine, Wroclaw University of Environmental and Life Sciences, Wroclaw 50-375, Poland, liliana.kiczak@upwr.edu.pl, michal.plociennik@upwr.edu.pl

^2^ Veterinary Center, Nicoalus Copernicus University in Torun, Torun 87-100, Poland, urszula.paslawska@umk.pl, robert.paslawski@umk.pl

^3^ Department of Internal Diseases and Clinic of Diseases of Horses, Dogs and Cats, Faculty of Veterinary Medicine, Wroclaw University of Environmental and Life Sciences, Wroclaw 50-375, Poland, kacper.nowak@upwr.edu.pl, urszula.paslawska@upwr.edu.pl

^4^Clinical Department of Anesthesiology and Intensive Therapy, Wroclaw Medical University, Wroclaw 50-556, Poland, waldemar.gozdzik@umw.edu.pl, barbara.adamik@umw.edu.pl, marzena.zielinska@umw.edu.pl, stanislaw.zielinski@umw.edu.pl

^5^ Department of Food Hygiene and Consumer Health Protection, Faculty of Veterinary Medicine, Wroclaw University of Environmental and Life Sciences, Wroclaw 50-375, Poland, jacek.bania@upwr.edu.pl, aleksandra.tabis@upwr.edu.pl

^6^ Department of Pathology, Faculty of Veterinary Medicine, Wroclaw University of Environmental and Life Sciences, Wroclaw50-375, Poland, marcin.nowak@upwr.edu.pl

^7^ Department of Anesthesia and Intensive Care, Karolinska Institutet, Danderyd Hospital, Stockholm 182-88, Sweden, claes.frostell@ki.se

*Corresponding author: Liliana Kiczak, Faculty of Veterinary Medicine, Department of Biochemistry and Molecular Biology, Wroclaw University of Environmental and Life Sciences, Norwida 31, 50-375 Wroclaw, Poland, phone +48 71 3205209,

e-mail: liliana.kiczak@upwr.edu.pl

**Supplementary Table 1**. Parameters of shock induced by endotoxin (heart rate, cardiac output, mean pulmonary arterial pressure, mean arterial pressure, central venous pressure, pulmonary capillary wedge pressure, temperature) and hypoperfusion (lactate concentration) measured at baseline, 4, 8, 12 and 20 hours of the study.

|  | treatment | baseline | 4h | 8h | 12h | 20h |
| --- | --- | --- | --- | --- | --- | --- |
| HR, beats per minute | Control | 70 ± 5 | 129 ± 13 | 134 ± 8 | 125 ± 8 | 121 ± 8 |
|  | iNO + HCT | 83 ± 8 | 105 ± 11 | 122 ± 12 | 124 ± 9 | 116 ± 10 |
| CO, L/min/m^2^ | Control | 3.4 ± 0.3 | 2.4 ± 0.1 | 3.4 ± 0.4 | 4.4 ± 0.5 | 4.9 ± 0.5 |
|  | iNO + HCT | 4.2 ± 0.3 | 2.4 ± 0.3 | 3.6 ± 0.4 | 4.2 ± 0.3 | 4.9 ± 0.4 |
| MPAP, mmHg | Control | 19 ± 1 | 38 ± 3 | 34 ± 3 | 29 ± 2 | 26 ± 2 |
|  | iNO + HCT | 18 ± 1 | 29 ± 2* | 27 ± 1* | 25 ± 1 | 21 ± 1 |
| MAP, mmHg | Control | 93 ± 3 | 90 ± 7 | 99 ± 6 | 87 ± 7 | 82 ± 3 |
|  | iNO + HCT | 102 ± 3 | 94 ± 6 | 101 ± 8 | 103 ± 8 | 92 ± 6 |
| CVP, mmHg | Control | 7 ± 1 | 6 ± 1 | 7 ± 1 | 8 ± 1 | 8 ± 1 |
|  | iNO + HCT | 6 ± 1 | 5 ± 2 | 8 ± 1 | 9 ± 1 | 8 ± 1 |
| PCWP, mmHg | Control | 9 ± 1 | 12 ± 3 | 10 ± 1 | 10 ± 1 | 13 ± 2 |
|  | iNO + HCT | 8 ± 1 | 10 ± 2 | 11 ± 1 | 11 ± 1 | 12 ± 1 |
| Temperature,°C | Control | 37.1 ± 0,3 | 38.7 ± 0.4 | 39.8 ± 0.5 | 40.3 ± 0.4 | 40.4 ± 0.4 |
|  | iNO + HCT | 36.9 ± 0.18 | 38.5 ± 0.2 | 39.1 ± 0.4 | 39.6 ± 0.4 | 40.2 ± 0.6 |
| Lactate, mmol/L | Control | 2.49 ± 0.39 | 4.19 ± 0.46 | 3.42 ± 0.61 | 2.15 ± 0.29 | 1.16 ± 0,13 |
|  | iNO + HCT | 2.21 ± 0.34 | 4.02 ± 0.71 | 2.89 ± 0.47 | 2.09 ± 0.56 | 1.55 ± 0.44 |

CO, cardiac output; CVP central venous pressure; HR, heart rate; MAP, mean arterial pressure; MPAP, mean pulmonary arterial pressure; PCWP, pulmonary capillary wedge pressure. Values are described as means ± standard deviation. Statistical significance was determined by the Mann-Whitney U test (* P <0.05 vs. control group).

**Supplementary Table 2.** Semi-quantitative histology data in pigs from iNO + HCT group (exposed to LPS with standard treatment + inhaled NO and IV hydrocortisone) and from control group (exposed to LPS with standard treatment).

|  | iNO + HCT group  (n = 12) | | | | Control group  (n == 11) | | | |
| --- | --- | --- | --- | --- | --- | --- | --- | --- |
| score | 0 | 1 | 2 | 3 | 0 | 1 | 2 | 3 |
| **Inflammatory infiltrates:** |  |  |  |  |  |  |  |  |
| Lymphocytes, N (%) | 0 | 10 (84) | 1 (8) | 1 (8) | 0 | 8 (73) | 2 (18) | 1 (9) |
| Monocytes, N (%) | 11 (92) | 1 (8) | 0 | 0 | 7 (64) | 4 (36) | 0 | 0 |
| Neutrophils, N (%) | 6 (50) | 3 (25) | 2 (17) | 1 (8) | 2 (18) | 5 (46) | 3 (27) | 1 (9) |
| Eosinophils, N (%) | 11 (92) | 1 (8) | 0 | 0 | 11 (100) | 0 | 0 | 0 |
| **Passive congestion,** N (%) | 2 (17) | 7 (58) | 3 (25) | 0 | 3 (27) | 6 (55) | 2 (18) | 0 |
| **RBC in the alveoli,** N (%) | 8 (66) | 2 (17) | 0 | 2 (17) | 6 (55) | 3 (27) | 1 (9) | 1 (9) |
| **Edema,** N (%) | 8 (67) | 4 (33) | 0 | 0 | 7 (64) | 4 (36) | 0 | 0 |

HCT, hydrocortisone; iNO, inhaled nitric oxide; RBC, red blood cells.

Morphologic changes were quantified using a scoring system of 0 to 3, with 0 being no change, 1 - mild lesion, 2 - moderate lesions, and 3 - severe lesions.

Inflammatory infiltrates: 0, lack or occasional infiltration of single cells; 1, a little amount of diffuse inflammatory cell infiltrate; 2, moderate amount of diffuse inflammatory cell infiltrate; 3, severe amount of diffuse inflammatory cell infiltrate.

Passive congestion: 0, no passive congestion; 1, blood-filled alveolar spaces in smaller area; 2, blood-filled alveolar spaces in larger area; 3, blood-filled alveolar spaces in large areas.

RBC in the alveoli: 0, no intra-alveolar RBC; 1, occasional finding of RBC in the alveoli; 2, moderate finding of RBC in the alveoli; 3, frequent finding of RBC in the alveoli.

Edema: 0, no edema; 1, fluid-filled alveolar spaces in smaller area; 2, fluid-filled alveolar spaces in larger area; 3, fluid-filled alveolar spaces in large areas.

**Supplementary Table 3.** Inflammatory mediators in pulmonary homogenates in a porcine endotoxemia model.

|  | control group  (n=11) | iNO + HCT group  (n=12) | p-value |
| --- | --- | --- | --- |
| IL-1β (pg/mg protein) | 43.45 ± 10.51 | 19.02 ± 2.93 | 0.029 |
| IL-6 (pg/mg protein) | 24.18 ± 6.96 | 12.37 ± 3.91 | 0.189 |
| TNF-α (pg/mg protein) | 20.36 ± 3.24 | 14.50 ± 2.06 | 0.346 |
| IL-8 (pg/mg protein) | 22.56 ± 5.77 | 14.14 ± 3.14 | 0.316 |
| IFN-α (pg/mg protein) | 0.17 ± 0.03 | 0.22 ± 0.04 | 0.525 |
| IFN-γ (pg/mg protein) | 240.43 ± 166.46 | 5.51 ± 1.38 | 0.328 |
| IL-10 (pg/mg protein) | 14.77 ± 0.77 | 12.66 ± 1.81 | 0.079 |
| IL-12p40 (pg/mg protein) | 40.55 ± 19.11 | 29.18 ± 8.98 | 0.927 |

HCT, hydrocortisone; iNO, inhaled nitric oxide; IL-1β, Interleukin 1 beta; IL-6, Interleukin 6; TNF-α, tumor necrosis factor alpha; IL-8, Interleukin 8; IFNα, Interferon alpha; IFN-γ, Interferon gamma; IL-10, Interleukin 10; IL-12p40, Interleukin 12p-40. Values are presented as means ± SEM. Statistical significance was determined by the Mann-Whitney U test.
